# Enhanced cellular therapy: revolutionizing adoptive cellular therapy

**DOI:** 10.1186/s40164-024-00506-6

**Published:** 2024-04-25

**Authors:** Meng-Yao Xu, Na Zeng, Chen-Qian Liu, Jian-Xuan Sun, Ye An, Si-Han Zhang, Jin-Zhou Xu, Xing-Yu Zhong, Si-Yang Ma, Hao-Dong He, Jia Hu, Qi-Dong Xia, Shao-Gang Wang

**Affiliations:** Department and Institute of Urology, Tongji Hospital, Tongji Medical College, Huazhong University of Science and Technology, No.1095 Jiefang Avenue, Wuhan, 430030 China

**Keywords:** Enhanced cellular therapy, Adoptive cellular therapy, Immunotherapy, Immune checkpoint inhibitor, PROTAC, Oncolytic virus

## Abstract

Enhanced cellular therapy has emerged as a novel concept following the basis of cellular therapy. This treatment modality applied drugs or biotechnology to directly enhance or genetically modify cells to enhance the efficacy of adoptive cellular therapy (ACT). Drugs or biotechnology that enhance the killing ability of immune cells include immune checkpoint inhibitors (ICIs) / antibody drugs, small molecule inhibitors, immunomodulatory factors, proteolysis targeting chimera (PROTAC), oncolytic virus (OV), etc. Firstly, overcoming the inhibitory tumor microenvironment (TME) can enhance the efficacy of ACT, which can be achieved by blocking the immune checkpoint. Secondly, cytokines or cytokine receptors can be expressed by genetic engineering or added directly to adoptive cells to enhance the migration and infiltration of adoptive cells to tumor cells. Moreover, multi-antigen chimeric antigen receptors (CARs) can be designed to enhance the specific recognition of tumor cell-related antigens, and OVs can also stimulate antigen release. In addition to inserting suicide genes into adoptive cells, PROTAC technology can be used as a safety switch or degradation agent of immunosuppressive factors to enhance the safety and efficacy of adoptive cells. This article comprehensively summarizes the mechanism, current situation, and clinical application of enhanced cellular therapy, describing potential improvements to adoptive cellular therapy.

## Background

**Fig. 1:**
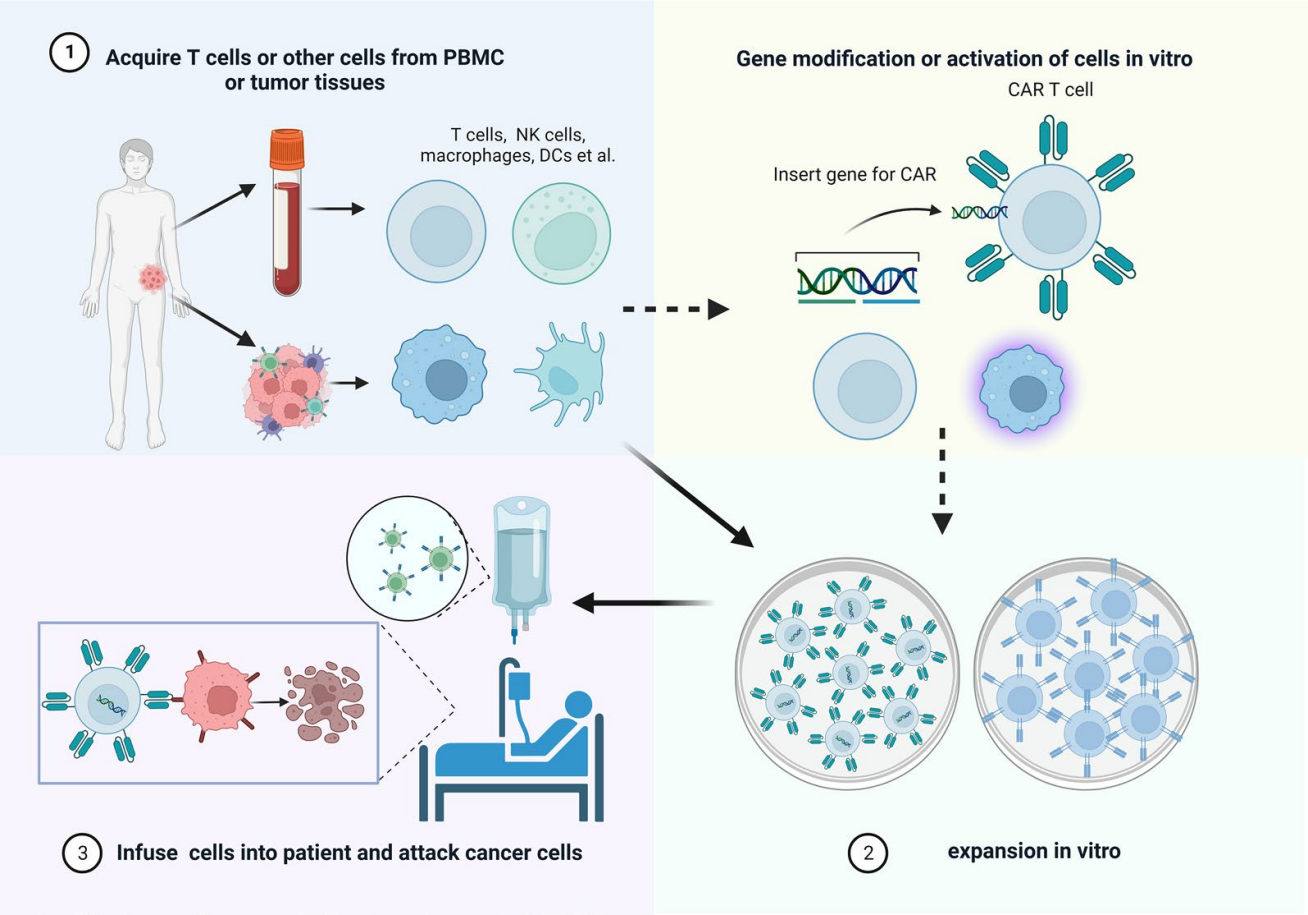
Steps of adoptive cellular therapy. (i) collection of immunoreactive cells from peripheral blood mononuclear cells (PBMC) or tumor tissues of patients; (ii) in vitro cell amplification; and (iii) cell transfusion back into patients to directly eliminate tumor cells or stimulate the immune response to damage the tumor. In some cases, ACT also requires genetic engineering or cell activation

As an emergent branch of immunotherapy, adoptive cellular therapy (ACT) employs lymphocytes and antigen-presenting cells (APCs). This modality can be briefly summarized into 3 steps (Fig. 1): (i) collection of immunoreactive cells from peripheral blood mononuclear cells (PBMC) or tumor tissues of patients; (ii) in vitro cell amplification; and (iii) cell transfusion back into patients to directly eliminate tumor cells or stimulate the immune response to damage the tumor. In some cases, ACT also requires genetic engineering or cell activation [1].

ACT originated from lymphokine-activated killer (LAK) cell therapy in the 1980s. LAK is a non-specific killer cell induced by IL-2 that is added to PBMC in vitro [2, 3]. Subsequently, researchers explored cytokine-induced killer (CIK) cell therapy, which is similar to LAK. A variety of cytokine or monoclonal antibodies are used in CIK, including IFN- γ, IL-15, and Anti-CD3mAb (OKT3) [4]. With the co-expression of CD3 + and CD56 + , CIK is also called NK cell-like T lymphocyte, which exhibits both specific tumor-killing ability and non-MHC limited cell-killing ability. Clinically, it is complemented by dendritic cells (DCs) to make up for the inadequate antigen presentation ability in CIK therapy [5]. In 1986, Rosenberg's team found a class of tumor-specific T cells in tumor tissues named tumor-infiltrating lymphocytes (TILs), which they applied as TIL therapy for clinical treatment. TIL therapy is described as the isolation of TILs from surgically resected tumor tissues, which are then transfused after massive expansion in vitro. TILs exhibit a tumor-killing activity about 50 times stronger than that of LAK [6]. In 2010, the first DC therapy, Provenge, was approved by the FDA to treat hormonal refractory prostate cancer [7, 8]. With the development of genetic engineering technology, T cell receptor-gene engineered T (TCR-T) cells, chimeric antigen receptor T (CAR-T) cells, chimeric antigen receptor natural killer (CAR-NK) cells and chimeric antigen receptor macrophages (CAR-M) have appeared gradually. In 2017, the United States Food and Drug Administration (FDA) approved the launch of Kymriah, the first CAR-T cell product, which pioneered the commercial era of CAR-T cells [9]. Furthermore, the first CIK cell therapy, ImmunCell-LC, was approved by the FDA in 2018 [10].

**Fig. 2:**
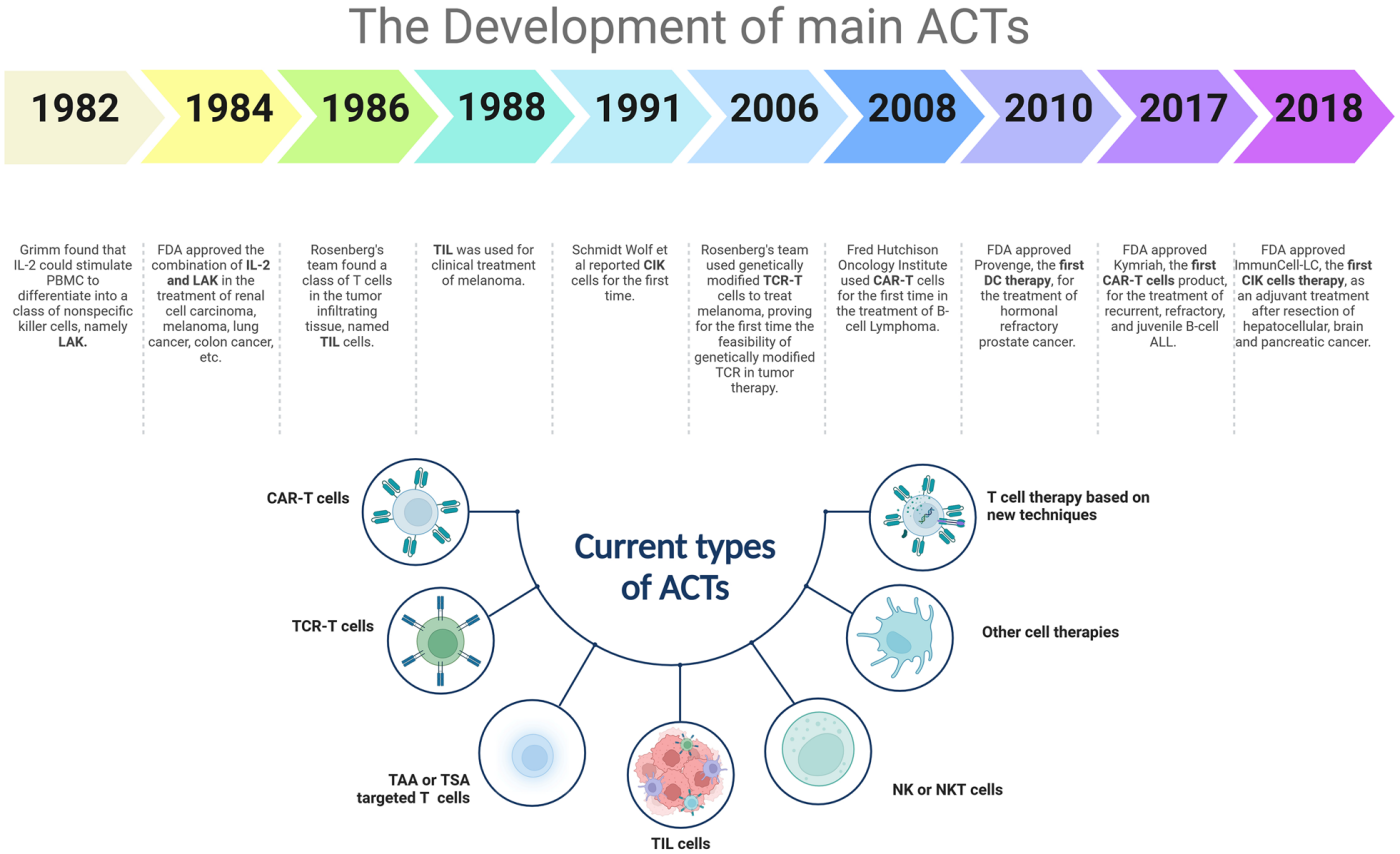
The Development of the main ACTs [15]. In 1982, Grimm found that IL-2 could stimulate PBMC to differentiate into a class of nonspecific killer cells, namely LAK. In 1984, the FDA approved the combination of IL-2 and LAK in the treatment of renal cell carcinoma, melanoma, lung cancer, colon cancer, etc. In 1986, Rosenberg's team found a class of T cells in tumor-infiltrating tissues, named TILs. In 1988, TIL was used for the clinical treatment of melanoma. In 1991, Schmidt Wolf et al. reported CIK cells for the first time. In 2006, Rosenberg's team used genetically modified TCR-T cells to treat melanoma, proving for the first time the feasibility of genetically modified TCR in tumor therapy. In 2008, Fred Hutchison Oncology Institute used CAR-T cells for the first time in the treatment of B-cell Lymphoma. In 2010, the FDA approved Provenge, the first DC therapy, for the treatment of hormonal refractory prostate cancer. In 2017, the FDA approved Kymriah, the first CAR-T cell product, for the treatment of recurrent, refractory, and juvenile B-cell ALL. In 2018, the FDA approved ImmunCell-LC, the first CIK cell therapy, as an adjuvant treatment after resection of hepatocellular, brain, and pancreatic cancer. Currently, ACTs include the following categories: CAR-T cells, TCR-T, autologous circulating T cells targeting tumor-associated antigen (TAA) or tumor-specific antigen (TSA), TIL, cell therapy derived from natural killer (NK) cells or natural killer T cells (NKT), T cell therapy based on new techniques (such as induced pluripotent stem cells, CRISPR or γ δ T cells), as well as treatments derived from other cell types (such as macrophages or stem cells)

In March 2020, global cancer cellular therapy included 1483 active agents [11]. Up to April 2021, 2073 cell therapies were under development in the global therapy pipeline [12]. Moreover, in April 2022, 2756 active cellular therapy drugs were under development in the global immunotherapy pipeline, of which 1432 were CAR-T cell studies, and more than half of the studies were still in the preclinical stage [13]. The 33 approved cellular therapy products worldwide include 21 kinds of stem cells and 12 kinds of immune cells (8 kinds of CAR-T cells, 3 kinds of DCs, and 1 kind of CIK) [14]. Based on the molecular mechanism, ACTs include the following categories (Fig. 2): CAR-T cells, TCR-T, autologous circulating T cells targeting tumor-associated antigen (TAA) or tumor-specific antigen (TSA), TIL, cell therapy derived from natural killer (NK) cells or natural killer T cells (NKT), T cell therapy based on new techniques (such as induced pluripotent stem cells, CRISPR or γ δ T cells), and treatments derived from other cell types (such as macrophages or stem cells).

Research on cellular therapy has exploded over the past three years, with CAR-T cells occupying the leading position. Regarding targets, CD19, BCMA, and CD22 are still the most commonly targeted proteins in hematological malignant tumors [16], while TAA, HER2, and mesothelin (MSLN) are the most commonly targeted proteins in solid tumors [13, 17]. There are currently 808 CAR-T cell-related clinical trials, of which 36 have been completed [18]. Although most studies have been conducted on hematological tumors, a growing number of CAR studies are being performed on solid tumors. In some small studies and case reports, CAR-T cell therapy has shown promising efficacy in various solid tumors, including mesothelioma, sarcoma, gastric cancer, and pancreatic cancer [19] (Table 1).

**Table 1:** Key enhanced cellular therapies in clinical trials

Title	Sponsors and collaborators	First posted	Phase	Conditions	Category	Trial number
Pembrolizumab in Patients Failing to Respond to or Relapsing After CAR T Cell Therapy for Relapsed or Refractory Lymphomas	Abramson Cancer Center of the University of Pennsylvania	January 8, 2016	phase 1/2	CD19 + Diffuse Large B-cell Lymphomas, Follicular Lymphomas, Mantle Cell Lymphomas	PD-1 inhibitor-enhanced CAR-T cell therapy	NCT02650999
A Study of Gene Edited Autologous Neoantigen Targeted TCR T Cells With or Without Anti-PD-1 in Patients With Solid Tumors	PACT Pharma, Inc	May 31, 2019	Phase 1	Solid Tumor	PD-1 inhibitor-enhanced TCR-T cell therapy	NCT03970382
RTX-240 Monotherapy and in Combination With Pembrolizumab	Rubius Therapeutics	May 4, 2020	phase 1/2	Solid Tumor, AML Adult	PD-1 inhibitor-enhanced erythrocyte cell therapy	NCT04372706
Study of IFN-α Combined With CAR-T Cell Therapy in Relapsed and Refractory Acute Lymphoblastic Leukemia (R/R-ALL)	The First Affiliated Hospital of Soochow University	September 1, 2020	Phase 2	B-cell Acute Lymphoblastic Leukemia	IFN-α-enhanced CAR-T cell therapy	NCT04534634
Cyclophosphamide Followed by Intravenous and Intraperitoneal Infusion of Autologous T Cells Genetically Engineered to Secrete IL-12 and to Target the MUC16ecto Antigen in Patients With Recurrent MUC16ecto + Solid Tumors	Memorial Sloan Kettering Cancer Center	July 15, 2015	Phase 1	Solid Tumor	Cyclophosphamide-enhanced TRUCK CAR-T cell therapy	NCT02498912
CD19 CAR-T Expressing IL7 and CCL19 Combined With PD1 mAb for Relapsed or Refractory Diffuse Large B Cell Lymphoma	Second Affiliated Hospital, School of Medicine, Zhejiang University	May 11, 2020	Phase 1	Diffuse Large B-cell Lymphoma	IL7 and CCL19-enhanced CAR-T cell therapy	NCT04381741
A Study of DeTIL-0255 in Adults With Advanced Malignancies	Nurix Therapeutics, Inc	November 4, 2021	Phase 1	Platinum-resistant Ovarian Cancer, Endometrial Cancer, Cervical Cancer	PROTAC-enhanced TIL therapy	NCT05107739
Binary Oncolytic Adenovirus in Combination With HER2-Specific Autologous CAR VST, Advanced HER2 Positive Solid Tumors	Baylor College of Medicine	November 14, 2018	Phase 1	Bladder Cancer, Head and Neck Squamous Cell Carcinoma, Cancer of the Salivary Gland, Lung Cancer, Breast Cancer, Gastric Cancer, Esophageal Cancer, Colorectal Cancer, Pancreatic Adenocarcinoma, Solid Tumor	Oncolytic Adenovirus-enhanced CAR-T cell therapy	NCT03740256

Following breakthroughs in ACT research in recent years, advances in immunotherapy are bringing better efficacy and fewer side effects. On the basis of cellular therapy, drugs or biotechnology can directly enhance or genetically modify cells to enhance the efficacy of adoptive cellular therapy, which is defined as enhanced cellular therapy. Unlike existing reviews, it delves into the concept of enhanced cellular therapy—a novel approach where drugs or biotechnologies not only bolster the killing ability of immune cells but also ensure their safety through methods such as chemotherapeutic drugs, immune checkpoint inhibitors (ICIs) / antibody drugs, small molecule inhibitors, immunomodulatory factors, proteolysis targeting chimera (PROTAC), oncolytic virus (OV), and tumor vaccines. What sets this review apart is its systematic exploration of ACT enhancements through various antineoplastic drugs or biotechnologies, marking a first in the field. It meticulously elucidates the mechanisms, current developments, and clinical applications of enhanced cellular therapy, shedding light on potential avenues for augmenting the efficacy of adoptive cellular therapy, and aiming to inspire innovative strategies for improving the outcomes of immunotherapy treatments (Table 2).

**Table 2:** Comparison of traditional ACT and enhanced cellular therapy

Therapy	Mechanism	Advantages	Disadvantages	Examples	Category
CAR-T cell	Through genetic engineering, T cells are activated and installed with tumor Chimeric Antigen receptor	1. Not MHC-restricted; 2. Strong signaling transduction through CD3ζ signaling pathway	1. Restricted to cell surface antigens; 2. Complicated preparation process in vitro for each patient	Carvykti, Kymriah	ACT
TCR-T cell	Using transgenic technology to install T cell receptors on T cells	1. Sensitive recognition; 2. Strong signaling transduction through integrated T cell signaling pathway	1. MHC-restricted; 2. Potential TCRs mismatch	Tebentafusp	ACT
TIL	Endogenous TIL was isolated from the resected tumor, amplified in vitro, and then injected back into the patient	1. Stronger tumor specificity; 2. More personalized	1. short duration of cell activity; 2. Long cultivation and amplification time	LN-145	ACT
PD-1 antibody-enhanced CAR-T cell therapy	ICIs such as anti-PD-1\ PD-L1 antibodies can block the brake of immune and enhance the anti-tumor activity of CAR-T cells	1. It prevent the failure of CAR-T cells and maintain its effector function 2. It makes up for the poor efficacy of CAR-T in solid tumors	1. Cannot block other immunosuppressive mechanisms, such as CTLA-4 2. drug resistance	αPD-1-mesoCAR-T cells	Enhanced cellular therapy
Interleukin-enhanced CAR-NK cell therapy	Cytokine drives the expansion and persistence of NK cells or other ATCs	1. Non-antigen specific manner without causing GVHD; 2. Highly cytotoxic effectors	1. Abnormal NK-cell proliferation or leukemia transformation lead by ectopic IL-15 production; 2. CRS	Cord blood NK cells engineered to express IL-15 and a CD19-targeted CAR	Enhanced cellular therapy
PROTAC-enhanced TIL	PROTACs degrade unwanted proteins in ACTs to enhance efficacy	1. Degradation of some inhibitors in TIL; 2. Increased T cell proliferation and reduced T cell failure	1. Relatively high toxicity; 2. Unknown consequences due to less research	DeTIL-0255	Enhanced cellular therapy
Oncolytic Virus-enhanced CAR-T	Strong immune response induced by oncolytic virus infection to enhance the efficacy of CAR-T cells	1. Promoted recruitment of CAR-T cells in TME by oncolytic virus; 2. More functional molecules expressed by modified oncolytic viruses	1. Selection of appropriate oncolytic virus; 2. Faster removal speed of oncolytic virus reduced by CAR-T	OV19t-enhanced CD19-CAR T	Enhanced cellular therapy

## ICIs/monoclonal antibody drugs-enhanced ACT

### Mechanism of ICIs

Tumor immunity generally consists of three steps: APCs recognize and present tumor cells, T cells activate and proliferate, and the effector T cells enter the tumor microenvironment (TME) to play an anti-tumor role. ACT focuses on the first two steps, while ICIs/ Monoclonal antibody drugs modulate the third step.

Acting as the brakes of the immune system, immune checkpoints prevent the overactivation of the immune system [20]. However, the immune escape mechanism of the tumor usually activates the immune checkpoint. Human immunity checkpoints mainly include: programmed cell death protein 1 (PD-1), cytotoxic T lymphocyte-associated protein 4 (CTLA 4), T cell immunoglobulin and mucin domain-containing protein 3 (TIM-3), V-domain Ig suppressor of T-cell activation (VISTA), lymphocyte activation gen (LAG)-3, signal regulatory protein alpha (SIRPα), T cell Ig and ITIM domain (TIGIT), B and T lymphocyte attenuator (BTLA), sialic acid-binding immunoglobulin-like lectin 7 (Siglec-7), and leukocyte Ig-like receptor subfamily B (LILRB) [21]. CTLA-4, also known as CD152, is a transmembrane receptor found on T cells, which shares the ligands of CD80 / CD86 with CD28. CTLA-4 binds to its ligand to trigger T cell anergy and participates in the negative regulation of the immune response [22]. Another immunosuppressive transmembrane protein PD-1 binds to PD-L1 to induce phosphorylation of the activation signal downstream of the T cell receptor and reduce its killing effect on tumor cells [23, 24]. Moreover, as a vital member of the immunoglobulin superfamily (IgSF), LAG-3 not only negatively regulates T cells, but is also co-expressed with PD-1 in TME [25]. Furthermore, tumor immunity can be enhanced by inhibiting the checkpoints that attenuate the immune function of the body. By the end of September 2022, only three types of ICIs were approved by FDA: CTLA-4 inhibitors, PD-1/PD-L1 inhibitors, and LAG3 inhibitors [26]. However, most patients cannot benefit from ICIs due to primary resistance arising from tumor specificity, or acquired resistance from long-term drug use [27]. In addition, the high level of immunosuppression around the solid tumor caused by immune suppressor cells such as regulatory T cells (Treg), bone marrow-derived suppressor cells (MDSCs), and M2 tumor-associated macrophages (TAM), leads to weak cellular therapy response [28, 29]. The production of local cytokines (including IL-4, IL-10, VEGF, and TGF β) promotes tumor growth and progress, and up-regulation of immune checkpoints also suppresses tumor immunity [28].

**Fig. 3:**
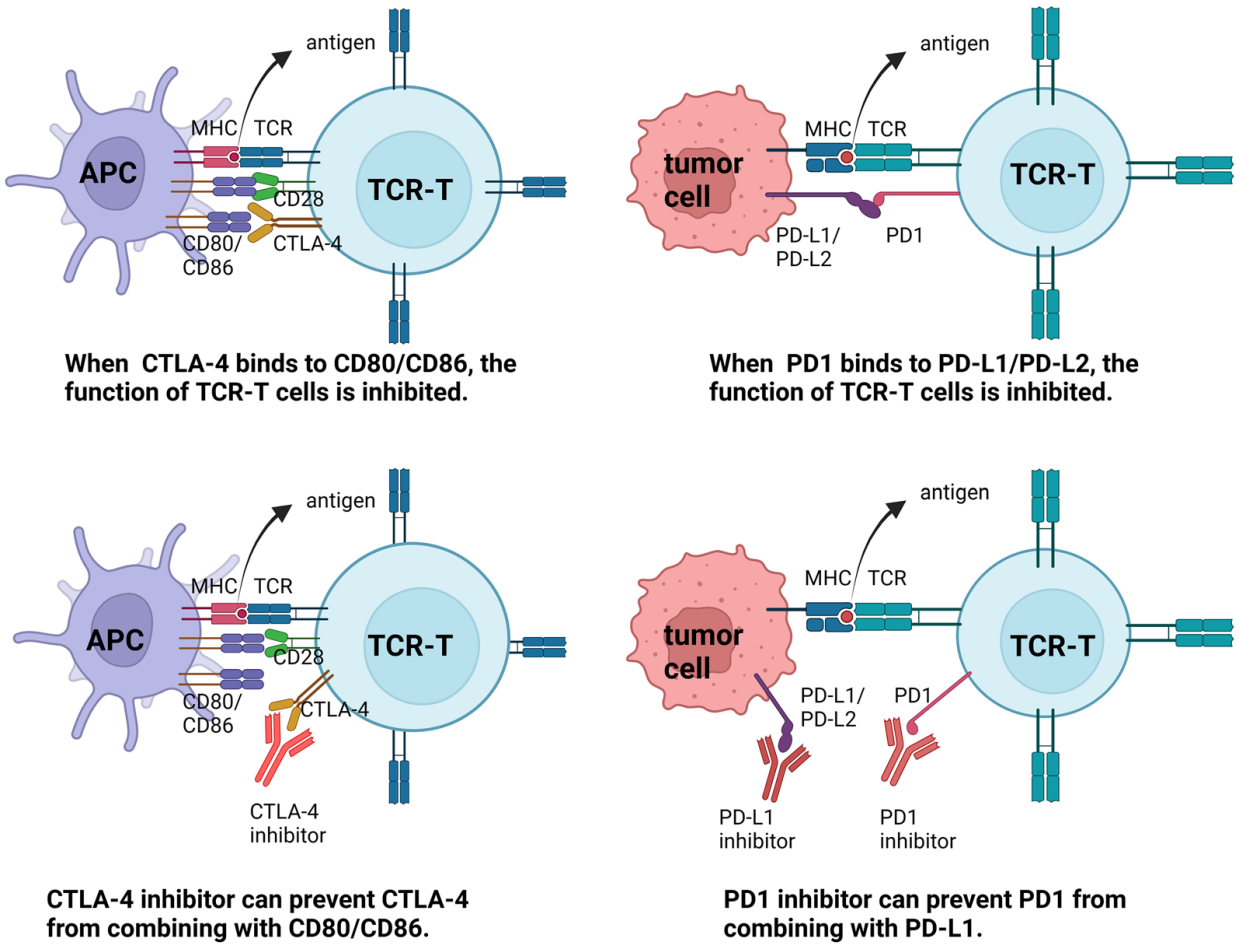
Mechanism of ICIs-enhanced ACT. ICIs are used to enhance the efficacy of ATC by combining with the corresponding targets to activate T cell anti-tumor immune function and reduce the secretion of inhibitory factors. When CTLA-4 binds to CD80/CD86, the function of TCR-T cells is inhibited. CTLA-4 inhibitors can prevent CTLA-4 from combining with CD80/CD86. The function of TCR-T cells is inhibited when PD1 binds to PD-L1/PD-L2. PD1 inhibitors can prevent PD1 from combining with PD-L1

In order to overcome the primary drug resistance of ICIs and improve the immunosuppression of ACT in TME, ICIs are used to enhance the efficacy of ATC to activate T cell anti-tumor immune function and reduce the secretion of inhibitory factors (Fig. 3).

### ICIs-enhanced CAR-T

Tumors gradually adapt to the treatment environment, and CAR-T cells may trigger the expression of PD-L1 on target cells and down-regulate CD28 costimulatory signals, weakening the efficacy of CAR-T cells. ICIs can block the PD-1/ PD-L1 axis, prevent the exhaustion of CAR-T cells, and maintain its effector function [30, 31]. In addition, the negative effect of the PD-1/ PD-L1 axis can be eliminated by modifying CAR-T cells by knocking out the PD1 coding gene *PDCD1* [32]. Studies have shown that blocking PD-1 with anti-PD-1 monoclonal antibodies significantly enhanced the expression of proliferation markers Ki-67, IFN γ, and granzyme B in CAR-T cells [33].

Furthermore, PD-1 inhibitors were found to result in the re-expansion of B cell mature antigen (BCMA) CAR-T cells in patients with myeloma [34]. The researchers introduced atezolizumab-based PD-L1-targeted CAR into T cells, and the combination of HER2-CAR T cells and PD-L1-CAR T cells showed significant benefits against breast cancer MCF-7 cells [35]. In mouse KP1233 (KP) tumor cells, CAR-T cells targeting receptor tyrosine kinase-like orphan receptor 1 (ROR1) cannot effectively invade tumors and are dysfunctional, while anti-PD-1 / PD-L1 can enhance CAR-T cell efficacy in cyclophosphamide (Ox) / oxaliplatin (Cy)-treated cancers [36]. A preclinical study demonstrated that CAR-T cell therapy armored to secrete a PD-1 blocking scFv could increase the ability of tumor-specific T cells to survive in vivo [37, 38]. The use of anti-CAIX CD28/4-1BB CAR-T cells to release anti-PD-L1 antibodies provides exciting new prospects for treating refractory clear cell renal cell carcinoma (ccRCC) [39]. Although ICIs-enhanced CAR-T cells revealed a satisfactory anticancer effect, other mechanisms of immunosuppression must be overcome in TME. In addition to PD-1, a variety of co-inhibitory receptors (such as TIM-3, LAG3, and TIGIT) are also expressed on depleted T cells [40].

### ICIs-enhanced TCR-T

TCR is an α/β chain or γ/δ chain heterodimer membrane protein that binds to the MHC antigen complex. TCR can target more antigens than CARs since MHC molecules can present peptide chains from the cell surface and intracellular proteins [41]. CAR-T cell exhaustion is particularly obvious in solid tumors [42] and the applicability of CAR-T cell therapy outside hematological malignant tumors is limited [43]. TCR-T cells are significantly more effective than CAR-T cells in treating most solid tumors due to sensitive recognition and robust signaling transduction through an integrated T cell signaling pathway [44, 45], especially in liver cancer, melanoma, and synovial cell sarcoma [46–48], although CAR-T is still used as a cell therapy strategy for neuroblastoma [49]. In addition, TCRs are naturally expressed in the human body and do not cause immune rejection [50]. However, the expression of TAAs in tumor cells is constantly changing, leading to drug resistance in some patients who receive ICIs and ATC, which hinders specific antigen-targeted therapy. Combination TCR-T therapy based on new antigens produced by tumor-specific mutation and ICIs can further improve the efficacy of immunotherapy [44, 51]. Removing the gene encoding PD-1 enhances the anticancer activity of TCR-T and improves the efficacy of cancer immunotherapy [52]. Moreover, some immunosuppressive factors such as TGF- β or PD-L1 can be blocked by the expression of chimeric switch receptor (CSR) on neoAg-specific TCR-T cells in TME [53]. Although TCR-T cell therapy has not been approved for the market so far, its unique advantages in solid tumors show promising applications.

### ICIs-enhanced TIL

TILs are composed of mixed cell types isolated from tumor samples, with T cells as the primary subtype [54]. Circulating lymphocytes in the blood penetrate the endothelial barrier and migrate to the tumor, where they are transformed into TILs. The quantity and quality of TILs are possible factors determining patient prognosis and treatment benefits [55]. The presence of immune checkpoint suppressors, such as PD-1, LAG-3, and TIM-3, accelerates the depletion of TIL. Antibodies against these checkpoint suppressors can partially restore the inhibited TILs response, exhibiting a more significant effect when used together [56].

A clinical trial at the Moffit Cancer Center, Tampa, US, demonstrated the feasibility of combining TIL with ICIs for the first time. 13 metastatic melanoma patients received standard TIL treatment combined with ipilimumab, and 1 patient showed complete remission (CR) 52 months after treatment [57, 58]. Moreover, a study of autologous TILs combined with nivolumab in the treatment of patients with advanced non-small cell lung cancer (NSCLC) (NCT03215810) showed that 2 patients had achieved and maintained CR after 1.5 years [59].

## Small molecule inhibitors-enhanced ACT

Small molecule inhibitors refer to organic compounds with a molecular weight of fewer than 1000 Daltons that can bind to targeted proteins, resulting in the reduction of protein biological activities [60]. According to the number and specificity of targets, they can be divided into two categories: multiple kinase inhibitors and selective inhibitors. Sorafenib and sunitinib are representative multiple kinase inhibitors, which exert anti-tumor activity by simultaneously targeting multiple cell kinases, such as VEGFR1, VEGFR2, KIT, and PDGFR- α targets point, and so on. In contrast, selective inhibitors have fewer targets and reduce the activity of a single component in the signal pathway by specifically antagonizing tumor cell targets. Representative drugs include erlotinib, gefitinib, and other EGFR inhibitors [61]. Trials involving small molecular inhibitors combined with ACT have been performed to enhance the efficacy of ACT.

A B-cell acute lymphoblastic leukemia (B-ALL) patient with positive Philadelphia chromosome relapsed after CD19-CAR-T cell therapy and was treated with a combination of blinatumomab (a bispecific T cell binding agent for CD19 and CD3) and ponatinib (a polytyrosine kinase inhibitor) and achieved a continuous remission of 12 months [62, 63]. Ibrutinib can be combined with other Bruton's tyrosine kinase (BTK) inhibitors to treat several kinds of B-cell malignant tumors. Furthermore, the combination of ibrutinib and CAR-T cells has shown significant efficacy in mouse xenograft models of CLL or ALL [64]. Combining ibrutinib and CD19-targeted CAR-T cells (CTL119) was also shown to be effective in patients with refractory CLL [65]. In addition, the combination of tyrosine kinase inhibitors (such as EGFR inhibitors) and ACT are being explored. For example, researchers at Baylor College of Medicine used TKI ibrutinib and dasatinib to improve anti-leukemic activity of CD5 CAR-T cells in patients with r/r T-ALL (NCT03081910). Nevertheless, the combined effect of small molecular inhibitors and ACT requires further experimental verification, and selecting appropriate tumor-specific target drugs from the large number of small molecular inhibitors is also challenging.

## Cytokines-enhanced ACT

### Mechanism of cytokines in ACT

The transformation from naive T cells to effector T cells requires three different signals, among which cytokine-mediated signal 3 is necessary for T cell proliferation and functional T cell memory development [66]. Cytokines, which include interleukin (IL), tumor necrosis factor (TNF), interferon (IFN), chemokine, colony-stimulating factor (CSF), growth factor, etc., participate in the activation, proliferation, differentiation, and survival of various immune cells [67, 68].

One of the limitations of ACT is the reduced efficacy against solid tumors due to the immunosuppressive TME conditions [69], including upregulated checkpoint receptors, inhibitory cytokines, variable chemokine expression profile, hypoxic environment, and abnormal tumor metabolism [70, 71]. It is a cunning strategy for improving the ACT effect that enhances T cell activation signals and changes the interaction between ACT cells and TME by transgene expression of cytokines or engineered cytokine receptors [72]. The fourth-generation CAR molecules (also known as TRUCK CAR) allow T cells to express secretory proteins such as cytokines IL-12, IL-18 and chemokines, etc. [73], while simultaneously expressing CAR molecules, which improves the invasive ability of CAR-T cells to resist the inhibitory TME conditions [74]. This review describes the regulation of cytokine expression and secretion to enhance the application of ACT in tumor therapy.

### Interleukin-enhanced ACT

#### γ chain cytokines and their receptors in ACT

The γ-chain co-receptor family of cytokines includes IL-21, IL-15, IL-9, IL-7, IL-4, and IL-2, which play a vital role in the differentiation and homeostasis of T cells, as well as in the proliferation, survival, and persistence of CAR-T cells [67, 75]. Currently, investigation of IL-4 and IL-9 in the context of ACT remains inadequate [76]. IL-2 is the only γ chain cytokine approved by the FDA for clinical trials, which can promote the expansion of adoptive immune cells in vivo in combination with adoptive TIL therapy by intravenous or subcutaneous administration [77]. Notably, activation-induced cell death (AICD) may be induced by long-term exposure to IL-2 [78], which mainly targets lymphocytes. IL-7 regulates homeostasis and promotes host defense by regulating the homeostasis of B progenitor cells, thymocytes, and peripheral mature T cells from human or mouse bone marrow [79]. In the ACT, the expression of CCL19 and IL-7 increases the infiltration of endogenous T cells, CAR-T cells, and DCs in tumors [80]. Its safety and efficacy in treating lymphoma, multiple myeloma, and solid tumors are under clinical testing and verification [76]. An animal experiment was conducted to investigate the efficacy of a long-acting genetically modified IL-7 in combination with CD19-targeted CAR-T cell therapy in mice with recurrent or refractory diffuse large B-cell lymphoma (DLBCL). This combination promoted the persistence, proliferation, and cytotoxicity of human CAR-T cells in mice, thereby significantly improving their survival rate [81]. The constitutive expression of IL-15 increases the anti-tumor activity of specific CAR-T cells targeting CD19, CLL-1, IL-13Rα2, GD2, and GPC-3 [82]. Among the γ-chain cytokines, IL-15 resulted in the highest persistence of CD19-CAR-T cells [83]. Since NK cells have shown remarkable cytotoxicity to tumor cells [84], CAR have not only been loaded on T cells but have also been expressed on the surface of NK cells by researchers [85]. Compared with non-transduced NK cells, cord blood-derived NK cells encoding CD19-CAR protein and IL-15 enhance the effector function of NK cells by cementing the Akt/mTORC1 axis and c-MYC signal transduction [86, 87]. In contrast to other γ-chain cytokines activating STAT5, IL-21 is inclined to activate STAT3 and mediate proliferation through PI3K and MAPK pathways [88]. Compared with IL-2, CAR-T expressing IL-21 reduces the end-effect differentiation of CD8 T cells, while different results of anti-tumor activity in vivo are shown in different studies [89, 90], so more clinical trials should be developed.

#### IL-12 family cytokines and their receptors in ACT

The IL-12 cytokine family includes IL-12, IL-23, IL-27, and IL-35. IL-23 and IL-12 are major proinflammatory/ pro-stimulatory cytokines that play a positive role in immune regulation [91, 92], while IL-27 and IL-35 play a negative role in anti-inflammation [93]. However, only IL-12 and IL-23 have been studied in ACT. Targeting both adaptive and innate immunity, IL-12 prolongs the survival and persistence of CAR-T cells, as well as recruits and activates effector cells to convert inhibitory TME into proinflammatory phenotypes [94]. IL-23 induces STAT3 activation, which improves the response of patients with chronic lymphoblastic leukemia [83, 95]. Recent studies have shown that engineered expression of IL-23 in CAR-T cells [96] may be an option to improve the function of CAR-T cells in solid tumors [76].

#### IL-1 superfamily cytokines in ACT

**Fig. 4:**
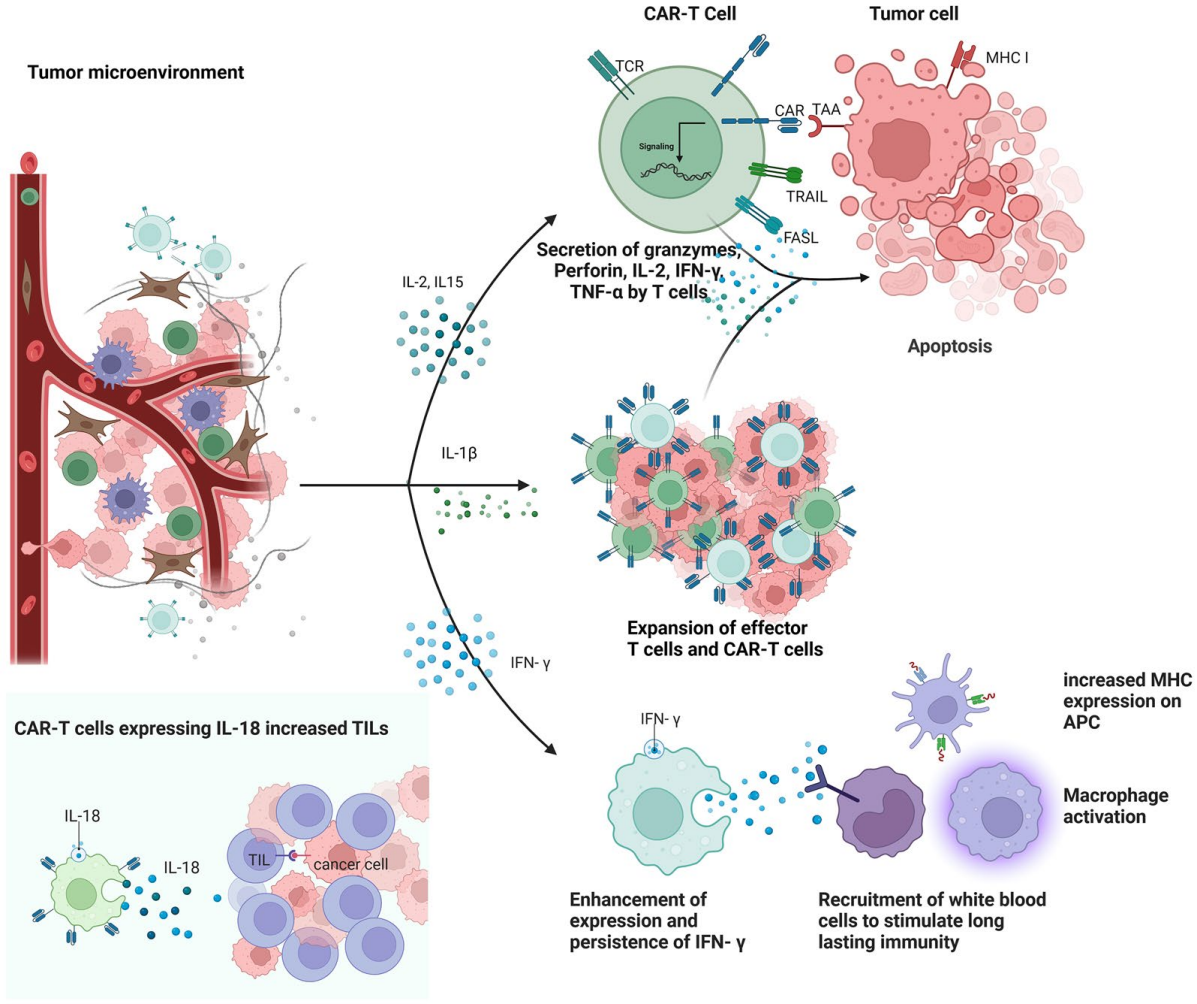
Roles of specific cytokines in ACT. IL-2 and IL-15 can trigger the secretion of granzymes, perforin, IL-2, IFN- γ, and TNF- α by CAR-T cells, promoting the apoptosis of tumor cells. IL-1β can promote the expansion of effector T cells and CAR-T cells. IFN- γ enhances the expression and persistence of IFN- γ, which can recruit white blood cells to stimulate lasting immunity, activate the macrophage, and increase the expression of MHC on APC

The IL-1 family of cytokines is a group of proinflammatory cytokines, including IL-1, IL-18, and IL-36 γ [97]. IL-1 plays a controversial role in tumorigenesis, with some studies proposing the induction of the pro-angiogenesis and metastasis-promoting factors expression [98], while anti-tumor activity has been reported [99]. The use of IL-1 β (injected s.c. in mice) significantly improved the efficacy of tumor regression by increasing the number and function of adoptive metastatic T cells in the tumor [100]. CAR-T cells expressing IL-18 attenuated the edematous toxicity induced by lower levels of TNF- α and IFN- γ, and increased TILs by reducing macrophage recruitment (Fig. 4) [101], resulting in an effective response in colon cancer models [102]. In a first-in-human study, IL-18 secreting autologous anti-CD19 CAR-T cells (huCART19-IL18) exhibit controllable toxic characteristics and satisfactory efficacy in patients with non-Hodgkin lymphomas relapsed or refractory to prior CAR-T cell therapy [103]. IL-36 includes IL-36 α, IL-36 β, and IL-36 γ [104], and IL-36 γ activates endogenous APCs and T cells, which contributes to the secondary anti-tumor response [105]. The engineered CAR-T cells expressing IL-36 γ can maintain stronger persistence and proliferation compared to unmodified CAR-T cells [76, 105].

### TNF-enhanced ACT

According to its origin and structure, TNF can be divided into TNF- α and TNF- β. The former is produced by mononuclear macrophages, which promote vascular permeability and blood coagulation and attract immune cells to prevent microbial infection from fighting tumors [106], while the latter is produced by activated T cells and becomes lymphotoxin [107]. TNF is essential to the anti-tumor function of cytotoxic T cells [108], assisting CAR-T cells in clinical treatment [109]. However, TNF also causes cytokine dysregulation and promotes tumor inflammation [110]. During CAR-T treatment, tumor necrosis factor-α (TNF-α) and IL1-β are secreted by activated bone marrow cells, which are the significant cytokines inducing endothelial activation [111] and aggravate the typical adverse reactions of CAR-T cell therapy, such as cytokine release syndrome (CRS) [112, 113]. Therefore, the application of TNF combined with ACT remains to be verified by clinical trials.

### INF-enhanced ACT

According to different receptor-binding, IFN can be divided into type I, type II, and type III. Type I IFN contains α, β, ω, and κ found in the human body. Type II IFN (immune IFN or IFN-γ) is produced by activated T cells and activated NK cells. Type III IFN, namely IFN-λ, includes IFN-λ1, IFN-λ2, IFN-λ3, and IFN-λ4 [114], and is responsible for triggering antiviral, anti-proliferation and pro-apoptotic responses [115]. Type I IFN can directly block cell cycle progression by inducing apoptosis, thereby promoting tumor clearance by stimulating immune cells to play an indirect anti-tumor effect [116]. Some clinical effects have been accomplished in treating breast cancer, melanoma, and renal cell carcinoma alone or in combination with IFN- α [117]. With a highly similar cellular signal transduction pathway to IFN-α, the newfound IFN, IFN- λ, shows less off-target effect in clinical practice [118]. IFN-λ enhances the killing effect of T cells and NK cells in melanoma, lung adenocarcinoma, and breast cancer [118]. Furthermore, Larson et al. demonstrated in glioblastoma tumors that the cytotoxicity of CAR-T cells in solid tumors is dependent on the tumor cell IFN-γ signaling pathway, which can activate endogenous T cells and monocytes/ macrophages in patients [119–121]. This is an example of the application of IFN-γ-enhanced ACT in solid tumors.

### Chemokine and chemokine receptor-enhanced ACT

The efficacy of CAR-T cell therapy in hematological tumors is partly due to the interaction of CAR-T cells in circulation. Therefore, the migration and infiltration of CAR-T cells are essential for their clinical effects in local solid tumors [122]. Co-expression of appropriate chemokine receptors can guide the homing of CAR-T cells to specific tumor cells [123], which combines the benefits of CAR-T and chemokine receptors. The modification of CC-chemokine receptor 4 (CCR4) in CAR-T cells had been identified to promote the migration of CAR-T cells expressing CCL17 and CCL22 to tumors, as well as the homing and anti-tumor activity in Hodgkin’s lymphoma xenograft model in mice [124, 125]. CAR-T cells expressing CCR2b improved the transport of tumor cells expressing CCL2, contributing to a tenfold increase in CAR-T cell infiltration into neuroblastoma [126]. CAR-T cells expressing CCL19 and IL-7 recruit APCs between tumors in the lung cancer model, increasing immune cell infiltration [127]. Whilding et al. proposed for the first time the use of C-X-C chemokine receptor (CXCR) 2 to direct CAR-T cell migration [128]. CXCR 1 and CXCR2 CAR-T cells maintain continuous tumor decline and immune memory in various tumor models such as glioblastoma, ovarian cancer, and pancreatic cancer [129, 130].

### Colony-stimulating factor, growth factor-enhanced ACT

Colony-stimulating factors include granulocyte-colony-stimulating factor (G-CSF) and granulocyte macrophage-colony-stimulating factor (GM-CSF). Mainly mediated by neutrophils and MDSC, G-CSF acts as a driving factor of hematopoietic stem cell (HSC) mobilization [131], while GM-CSF can promote the proliferation and differentiation of bone marrow progenitor cells to form granulocyte and macrophage colonies in vitro [132]. Many studies have confirmed the tumor-promoting effect of G-CSF [133], and the association between tumor-derived G-CSF and poor prognosis [134]. However, only a limited number of preclinical studies have investigated G-CSF and immunotherapy, and conclusions on whether G-CSF impairs T cell activity cannot be drawn yet. Macrophage colony-stimulating factor-1 receptor (CSF-1R) is expressed in CAR-T cells and generates a response to CSF-1, which enhances the proliferation of CAR signal transduction [124, 135]. Sterner et al. have demonstrated that the GM-CSF neutralizing antibody lenzilumab can enhance the anti-tumor activity of CAR-T cells and significantly reduce the severity of CRS and neuroinflammation (NI) in patients with acute lymphoblastic leukemia (ALL) treated with CAR-T cells targeting CD19 [136].

TGF- β induces epithelial-mesenchymal transition (EMT) in cancer cells and promotes angiogenesis, which is the main immunosuppressive regulatory factor in TME and promotes cancer development [137]. Prostate cancer is characterized by a lack of proinflammatory cytokine production and T cell infiltration and is regarded as a cold tumor in immunology [138]. Knockout of TGF- β signal from CAR-T cells has been shown to enhance their proliferation and anti-tumor activity in a PSMA-specific advanced prostate cancer mouse model [139]. Moreover, clinical trials are being developed in patients with refractory castration-resistant metastatic prostate cancer (NCT03089203).

## PROTAC-enhanced ACT

### The structure and principle of PROTAC

Traditional small molecular inhibitors play a therapeutic role by interfering with the function of proteins, while protein-targeted degradants (PTDs) work through proteasome degradation of pathogenic target proteins, demonstrating higher selectivity and efficacy [140]. Among the current targeted protein degradation strategies, PROTAC has gained researchers’ attention. The concept of PROTAC was first proposed by Crews et al. in 2001, who successfully designed and synthesized the first batch of PROTAC bifunctional molecules for the degradation of methionine aminopeptidase 2 (MetAP-2) [141]. PROTAC molecules consist of three parts: a target protein-binding ligand (POI ligand), a E3 ubiquitin ligase-binding ligand (E3 ligand), and an intermediate linker [142]. When E3 ubiquitin ligase is activated, the target protein is labeled by ubiquitin and then degraded by the ubiquitin–proteasome system (UPS) [143]. Notably, PROTAC is dissociated to participate in a new round of degradation [144].

Both small molecular inhibitors and macromolecular antibodies employ “occupancy-driven” mechanisms, during which they occupy the active site of the target protein continuously to block its function [145]. More than 85% of the known disease-related proteins lack targeted drugs [146], which might be attributed to their intracellular or intranuclear distribution, out of reach of macromolecular antibodies [147]. Furthermore, their relatively smooth surface provides no “pocket” for small molecules to attach [148]. In contrast, PROTAC features an “event-driven” mechanism, in which it only provides the binding activity and triggers the combination of target proteins to E3 enzymes to degrade pathogenic proteins [146]. This method represents a new approach to targeting proteins that were traditionally thought to be unreachable (undruggable targets). The development of PROTAC focuses on inhibiting the proliferation and migration of tumor cells, as well as promoting tumor senescence and apoptosis. In addition to tumors, PROTAC is also effective in treating autoimmune diseases and neurodegenerative diseases [149], such as KT-474, an oral bifunctional small molecule IRAK4 degrading agent, which is used for the treatment of atopic dematitis (AD) or hidradenis suppurativa (HS) (NCT04772885). However, PROTAC is a triplet with considerable molecular weight, and is currently limited by its poor water solubility, oral bioavailability, membrane permeability, and difficulty of synthesis. To date, the research and development of PROTAC can be summarized as the search for innovation regarding its three components, among which the popularity of the E3 ligand is increasing. More than 600 ubiquitin ligases encoded by the human genome have been identified [150], but the main E3 ligases applied to PROTAC are limited to Cereblon (CRBN), von Hippel-Lindau (VHL), inhibitor of apoptosis protein (IAP), and Mouse Double Minute 2 (MDM2) [149]. Exploring more specific POI and E3 ligase in PROTAC is one of the directions in the future.

### The progress of PROTAC

The molecule PROTAC-1 ushered in an era of the first generation of peptide-based PROTAC molecular. Ubiquitin ligase SCF ^β−TRCP^ (SKP1-CUL1-F-box) is a part of PROTAC [141]. Based on SKP1-CUL1-F-box, scientists have applied PROTAC to achieve targeted degradation of breast cancer-related estrogen receptor (ER) and prostate cancer-related androgen receptor (AR) [151]. Subsequently, a peptide from hypoxia-inducible factor 1 subunit alpha (HIF1 α), named Fu-SMPI [152], was found to bind to VHL E3 ligase, and a cell-penetrating PROTAC that can degrade a series of POIs was then designed. Due to containing the peptide ligand of E3 ligase, the peptide group PROTAC is not a complete small molecular structure, and the large molecular weight is easily recognized by the immune system, causing antibody production [153]. In addition, low activity and unsatisfactory cell membrane permeability promote the development of small molecular PROTACs.

Crew’s team reported the first small molecule PROTAC in 2008, called SARM-nutlin PROTAC, which could induce the degradation of AR. In SARM-nutlin PROTAC, the MDM2-p53 protein–protein interaction (PPI) inhibitor nutlin-3a acts as the E3 ligand, and the non-steroidal androgen receptor ligand (SARM) flutamide derivative is the AR ligand [154, 155]. The BET protein family includes bromine domain protein (BRD)2, BRD3, BRD4, and testis-specific BRD (BRDT) [156]. In addition, small molecule inhibitors JQ1 and OTX015 were manufactured to induce apoptosis of BRD4-dependent cancer cells by acting on BRD4, a member of the BET protein family [157]. ARV-771 and ARV-825 were synthesized based on JQ1 and OTX015, effectively reducing the levels of BRD4 [158]. Furthermore, ARV-825 significantly inhibited tumor growth in an ALL xenograft mouse model [159]. Considering their effects on signal transduction and cell cycle regulation of tumor cells [160], protein kinases can inspire the POI choice of PROTAC design to inhibit abnormal protein kinase activity and treat malignant tumors. For example, PROTACs synthesized with bosutinib and dasatinib as BCR-ABL ligands can degrade BCR-ABL [161], facilitating the treatment of chronic myeloid leukemia (CML) caused by BCR-ABL structural proteins. PROTAC targeting epidermal growth factor receptor (EGFR) has been used to study tumor cells such as non-small cell lung cancer [162].

**Fig. 5:**
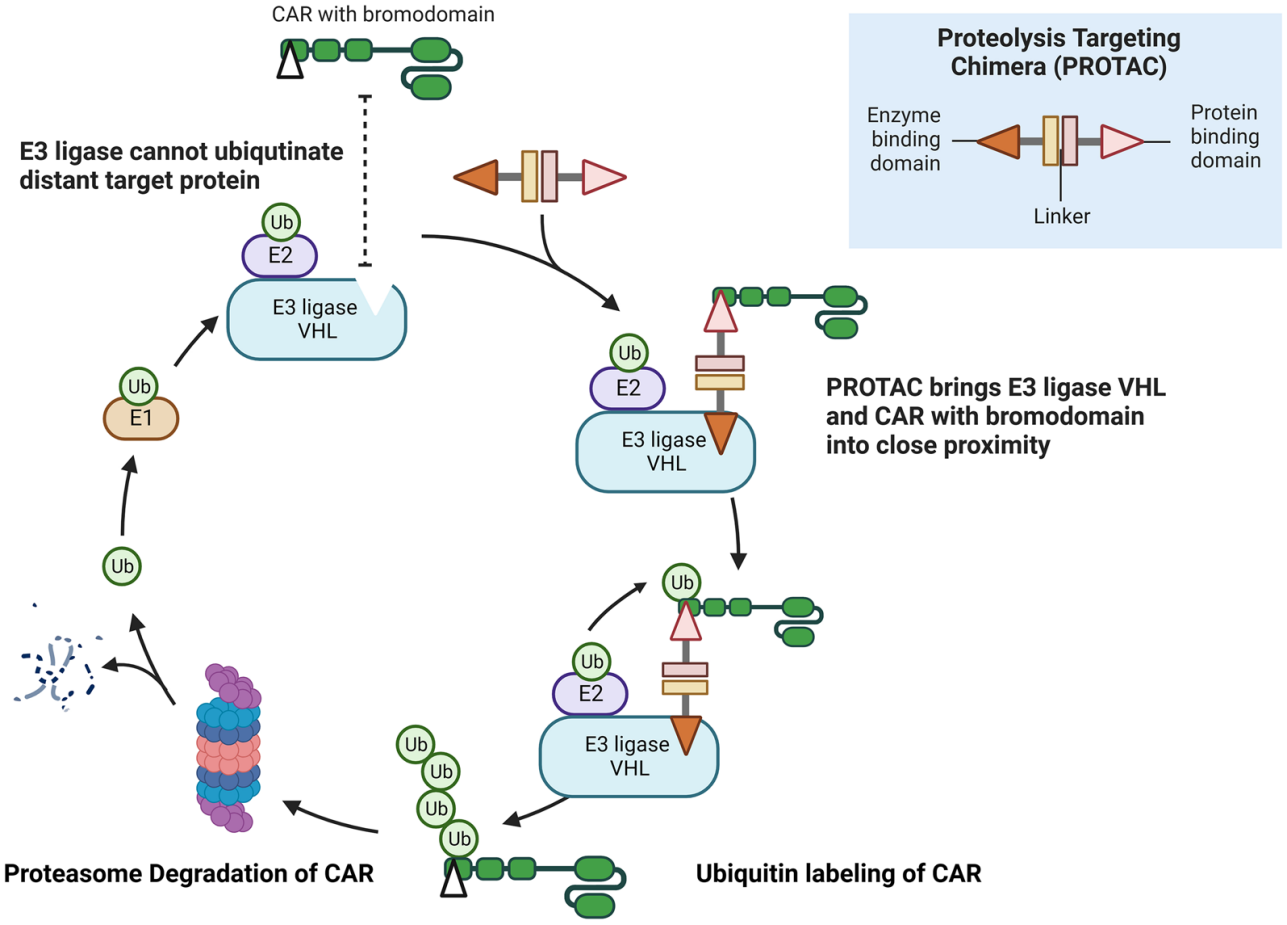
Degradation of CAR by PROTAC. PROTAC consists of three parts, namely the enzyme binding domain, linker, and protein binding domain. When E3 ubiquitin ligase (VHL) is activated, the target protein (CAR with bromodomain) is labeled by ubiquitin and then degraded by the ubiquitin–proteasome system (UPS)

Due to catalytic activity, the main disadvantages of PROTAC are non-cancer specificity and relatively high toxicity [140]. Light-controllable PROTAC, also known as the third-generation controllable PROTAC, was developed to overcome this issue. It contains two forms and triggers target protein degradation by UVA or visible light [140]. Photocaged PROTAC can only be transformed from its inactive to active structure, while photoswitchable PROTAC can reversibly change between active and inactive structures [163]. Photodynamic therapy (PDT) has been widely explored in prostate cancer and non-small cell lung cancer [164, 165], with a variety of light-controllable PROTACs reported to successfully degrade various targets, such as BRD4, FKBP12, IKZF1/3, ALK, BTK, and so on [163]. The characteristic of low toxicity, high temporal and spatial resolution of light-controllable PROTAC [166, 167] make up the application of PROTACs in tumor therapy (Fig. 5).

### PROTAC technology can enhance the safety of CAR-T cells

CRS is characterized by high fever, hypotension, myalgia, and even respiratory failure, which are considered adverse reactions associated with CAR-T cell therapy [168]. Different small molecules were developed in an attempt to minimize treatment-related toxicity while enhancing the therapeutic efficiency of CAR-T cells. The small molecule safety switches of CAR-T cells are divided into two types: “on” and “off” switches. PROTAC, as one of the “off” switches, regulates the stability of CAR through the UPS [169]. For example, ARV-771 inhibited the AR pathway and caused tumor regression in a castration-resistant prostate cancer (CRPC) mouse xenograft model [158, 170]. The PROTAC molecules ARV771 or ARV825 with bromine domain (BD) as POI and VHL or CRBN as ubiquitin ligase ligand can degrade the CAR protein expressing BD structure [158, 170, 171]. PROTAC can regulate the efficacy of CAR-T and inhibit tumor growth by turning off the “CAR” protein instead of degrading CAR-T cells. Moreover, adding BD to the CAR protein does not interfere with the secretion of cytokines by the original CAR-T cells and preserves the function of killing target cells. In addition, PROTAC technology enables the “reversible” control of CAR-T cells and improves the safety of CAR-T cell therapy [172].

### PROTAC technology-enhanced TILs

In addition to CAR-T, PROTAC is also combined with TILs. Casitas B-lineage lymphoma proto-oncogene B (CBL-B) is an important negative regulator of immune activation [173]. Clinical studies have shown that CBL-B-mediated ubiquitin prevents multidrug resistance (MDR) of different cancers during chemotherapy [174]. Nurix Therapeutics Corp. has proposed a therapy called DeTIL-0255 that employs NX-0255, a targeted CBL-B degrader, to enhance the killing ability of TILs injected back into patients’ bodies [175]. One phase 1 multicenter, open-label oncology study (NCT05107739) has been conducted by Nurix Therapeutics Corp. in December 2021 to evaluate the safety and tolerance of DeTIL-0255 in advanced malignant tumors (platinum-resistant ovarian cancer, endometrial cancer, cervical cancer) in adults. It was the first combination of PROTAC technology and TIL, presenting an innovative idea for PROTAC-enhanced ACT.

## OV-enhanced ACT

### Overview of OV therapy

Oncolytic virotherapy is an essential branch of tumor immunotherapy and has broad application prospects in the field of tumor therapy. Since the middle of the nineteenth century, many reported cases have shown that tumor regression was accompanied by natural virus infection [176]. For example, a woman with chronic leukemia was inadvertently infected with the influenza virus, which resulted in leukemia symptomatic relief and disease remission [177]. At the beginning of the twentieth century, the idea of using viruses to treat tumors was first put forward, introducing OV into tumor immunotherapy. However, little attention was paid to oncolytic therapy due to its strong immune response and complications caused by natural OVs, until scientists successfully modified the virus gene [176]. In October 2015, the FDA approved the first OV drug, a genetically modified HSV-1 named Imlygic (Talimogene laherparepvec; T-VEC), to treat melanoma by intratumoral injection [178]. Up to the first half of 2021, four OV drugs were approved in the world: RIGVIR (ECHO-7 virus) [179], Oncorine (H101) (recombinant human adenovirus type 5) [180], T-Vec (herpes simplex virus), and Delytact (Teserpaturev/G47 virus) [181].

OVs destroy tumor cells directly and stimulate the body to produce an anti-tumor immune response [182]. OVs are divided into two categories, including natural viruses such as reovirus, enteroviruses, Newcastle disease virus (NDV), and measles virus (MV), etc., and genetically engineered viruses such as herpes simplex virus (HSV) and adenovirus [183]. These modifications enhance the targeting ability, selective replication, cleavage potential of viruses, and host anti-tumor immunity [182, 184]. However, systemic administration of OVs might be detected and cleared by the immune filtration system in blood circulation [185], so the treatments are more effective as intratumoral administration. Although the curative effect of OV alone is not ideal [186], its advantages of broad anticancer spectrum and drug safety make the current strategy allow for combination therapies [186], such as ICIs (PD-1/PD-L1), ACT (CAR-T cells [187], CAR-NK cells [188]), mesenchymal stem cells (MSCs) [189] and neural stem cells (NSC) [190, 191]. When taken up by tumor-infiltrating immune carrier cells, OVs are successfully protected and preferentially transported to tumors [192], also improving the systemic delivery of OVs.

### OV enhances the immunogenicity of ACT

ACT shows excellent efficacy in the treatment of hematological cancers, whereas serious limitations exist in most solid tumors [193]. The therapeutic strategy for immune cold tumors focuses on enhancing T cell response and removing immunosuppressive coinhibitory signals [194]. In tumor immunity, OV can promote the activation of T cells [195], and ACT can promote the amplification of T cells [127]. Therefore, promoting activated expanded T cell transport and infiltration has been proved to drive T cells into the tumor [196]. Using OVs as initiation therapy can be a potential therapeutic strategy for “heating” cold tumors [197]. OVs exhibit anti-tumor activity by producing inflammatory stimulators such as type I IFN in TME, which provides the third signal stimulation needed for effector T cell activation [198]. On the other hand, OVs also cause immunogenic cell death (ICD) by infecting cells and releasing TAAs, pathogen-associated molecular patterns (PAMPs) [199], and internal damage-associated molecular patterns (DAMPs) [200]. Local delivery of cytokines in OVs is a more appropriate and safer treatment for the combination with CAR-T cells [201]. In addition, OVs express bispecific T cell conjugate (BiTE) to stimulate T cell-mediated bystander effect and kill tumor cells without OV infection, promoting the infiltration of CAR-T cells [201]. Furthermore, OVs can also be combined with TCR-engineered T cells to address the urgent need for better curative effect on patients with advanced solid tumors [188]. OVs promote the accumulation of modified adoptive cells and autoimmune cells in the tumor area, resulting in a greater immune effect than monotherapy.

**Fig. 6:**
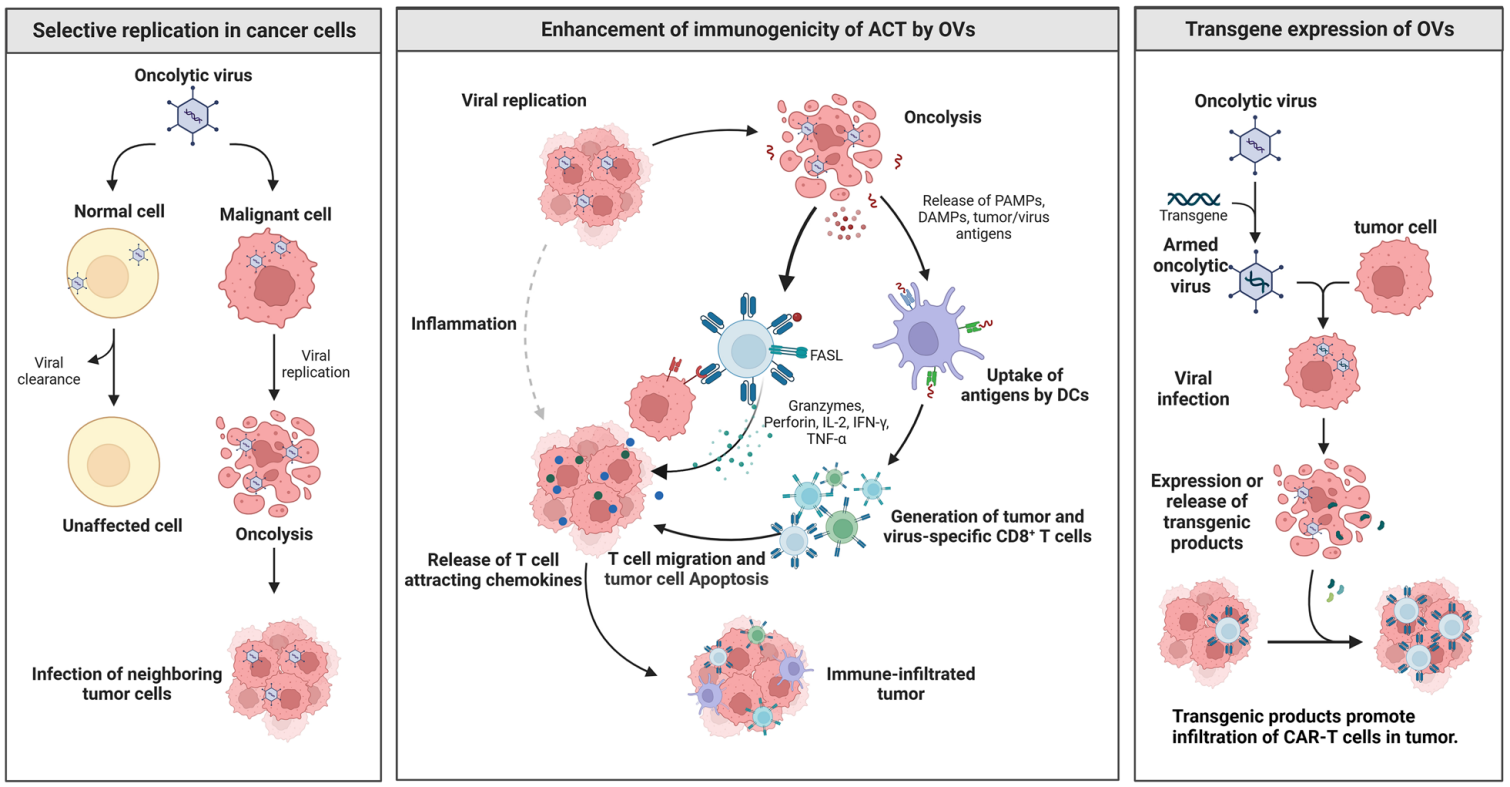
OV-enhanced ACT. selective replication in cancer cells: OVs are cleared from normal cells, but replicate in malignant cells, leading to cleavage and releasing more OVs to infect the neighboring tumor cells. Enhancement of immunogenicity of ACT by OVs: More OVs released by lytic tumor cells promote the release of PAMPs, DAMPs, and tumor/virus antigens, and then induce uptake antigens by DCs, generation of tumor and virus-specific CD8 + T cells. Meanwhile, OVs trigger CAR-T cells to release more granzymes, perforin, IL-2, IFN-γ, and TNF-α, which synergistically promotes the release of T cell attracting chemokines and T cell migration and tumor cell apoptosis. Transgene expression of OVs: When tumor cells are infected with armed OVs, transgenic cell products are expressed and released to enhance the infiltration of CAR-T cells in the tumor

Different combinations of OV and tumor immunotherapy (checkpoint blocking therapy, CAR-T cell therapy, BiTE, and cancer vaccine) can be applied to personalized cancer immunotherapy [202], while combination with OV has been shown to enhance the anti-tumor activity of adoptive immune cells [188] (Fig. 6).

### OV-enhanced CAR-T

In order to strengthen the recognition ability of adoptive cells, Priceman et al. have genetically engineered OV to form OV19t, which can enter tumor cells and force the expression of CD19 protein on the cell surface. Scientists were then able to identify and attack these solid tumors using CAR-T cells targeting CD19. OV-enhanced CD19-CAR-T therapy has been successfully implemented in triple-negative breast cancer cell lines, prostate cancer, ovarian cancer, pancreatic cancer, head and neck cancer, and brain tumor cells [203].

Siri et al. established the mouse melanoma model with normal immune function, which expressed ovalbulin, providing a rationale for treatment with OT-1T-cells. And they combined the intratumoral injection of oncolytic adenovirus with activated OT-I T cells in vitro, resulting in increased endogenous CD8 T cells [204]. In terms of neurological tumors, researchers from the University of Tokyo and Nagoya University in Japan have demonstrated for the first time that the combination of CAR-T cells and OVs has significantly prevented the growth of glioblastoma (GBM) and improved the survival rate of mice [205].

In addition, some OVs have been designed to provide immunostimulatory cytokines to promote the migration of CAR-T cells in solid tumors. The oncolytic adenovirus Ad-mTNFα-mIL2 expresses TNF- α, and IL-2 and was used in combination with mesothelin-redirected CAR-T (meso-CAR-T) cells to treat human pancreatic ductal adenocarcinoma (PDA) xenotransplantation immunodeficient mouse. Additionally, increased infiltration of CAR-T cells and host T cells was observed in PDA, accompanied by DC maturation and M1 polarization of macrophages [206]. The efficacy of oncolytic adenovirus expressing IL-7 combined with CAR-T cells targeting B7H3 was stronger than that of monotherapy [207].

Viral infection results in increased expression of PDL1, CTLA4, and other immune checkpoints [208]. In addition to cytokines, OVs modified with ICIs also perform a great enhancement effect on CAR-T cell therapy. Tanoue et al. found that in the prostate cancer xenotransplantation model, CAd-VECPDL1, a type of OV expressing PD-L1 blocking small antibodies, boosted the anti-tumor activity of CAR-T cells targeting HER2 [209]. Moreover, CAdTrio not only expressed cytokines (IL-12) and immune checkpoint blockers (PD-L1Ab) but also integrated the oncolytic adenovirus targeting CD44 variant 6 (CD44v6)-specific BiTE, which was combined with HER2-specific CAR-T cells to improve tumor control significantly [210].

### OV-enhanced TCR-T, CAR-NK, and TIL

However, few studies have investigated the combination of OV and TCR-T cells. Recently, Lu Yong’s team used myxoma virus (MYXV) to infect CAR-T and TCR-T cells to form CAR-T^MYXV^ and TCR-T^MYXV^ cells, inducing a new form of cell death termed autosis. This process enhanced the clearance of tumor cells by destroying tumor cells near the target through the “side killing effect” [211]. Furthermore, intravenous injection of CAR-T^10%MYXV^/ MART-1 T^10%MYXV^ in a mouse model of ovarian cancer and melanoma cured both tumors and showed no recurrence [211]. As a target of TCR-T cells, OVs enhance the heterogeneity of solid tumors and weaken the inhibitive immune microenvironment [212].

NK cells have obvious advantages over αβ T cells in immunotherapy as they do not trigger graft-versus-host disease (GvHD) [213]. In an orthotopic GBM mouse model [214], the combination of oncolytic virus expressing IL15/IL15Rα and EGFR-CAR-NK cells causes a strong anti-tumor response. Moreover, in a mouse colon cancer model [215], the combination of CCL5-modified oncolytic VACV and CCR5-overexpressing NK cells shows greater infiltration of NK cells in TME and more satisfactory efficacy than monotherapy.

The immunogenicity of most human solid cancers is poor, with low TIL counts in tumor tissues [216]. Therefore, OVs might be used to enhance the effect of tumor-specific TILs. Mathilde et al. established an MC38 murine colon tumor model with low immunogenicity and low levels of inflammatory infiltrate. Local injection of oncolytic poxvirus led to rapid accumulation of tumor-specific TIL in the tumor tissue, as well as longer survival in MC38-bearing mice [217].

In summary, OV is designed to boost the recognition, infiltration, migration, and activation of ACT cells, which is a promising approach to overcoming the challenges of ACT therapy in solid tumors.

## Challenges and prospects

### Unexpected combined adverse reactions

Different treatment combinations can be used to compensate for various shortcomings, but they generally lead to increased overall systemic toxicity. For example, as the most common adverse reaction of CAR-T cell infusion, CRS can cause multiple organ toxicity and disseminated intravascular coagulation (DIC) [218]. Besides, IFN-γ, IL-6, IL-10, IL-15, and GM-CSF are associated with another unique toxicity of CAR-T therapy, immune effector cell-associated neurotoxicity syndrome (ICANS) [219]. It remains to be verified whether cytokines can promote the occurrence of CRS or ICANS while enhancing immune activation. Moreover, the IL-15/IL-15R α complex produced by genetically-edited OVs further activates other immune cells, which aggravates inflammation or poisoning in patients [214]. While the most frequently observed long-term adverse effects in ACT to date include B cell depletion, hypogammaglobulinemia, reduced blood cell counts, and infections [220], the FDA has disclosed a more grave concern: all approved CAR-T cell therapy products (Yescarta, Tecartus, Kymriah, Breyanzi, Abecma and Carvykti) targeting BCMA or CD19 are associated with a significant risk of severe secondary T-cell lymphomas. These adverse reactions pose significant challenges to the regulatory of ACT. The FDA reported incidents of T-cell lymphomas following treatment with all six approved products on July 9, 2023. Although the overall benefits to patients from the aforementioned products outweigh the potential risks, the need for further regulatory scrutiny remains.

### Uncertain mode of medication

Moreover, several challenges remain concerning drug administration. In OV-enhanced cellular therapy, the administration protocol of intratumoral injection and intravenous injection remains controversial. Intratumoral injection of OVs reduces the consumption by the autoimmune system in circulation, but intravenous injection promotes the mobilization of immune cells in circulating blood before reaching the tumor [188, 221]. Furthermore, whether the drug or biotechnology be used only after ACT resistance or at the beginning of treatment is still unknown. In addition, the dosage might potentially need to be modified in monotherapy compared to combination therapy to avoid potential side effects. The mode of administration should be adjusted based on prognostic parameters to formulate therapy standards.

### Selection of novel construction or combination

Recent studies have focused on engineering the structure of adoptive cells to enhance their anti-tumor activity. For example, knocking down the PD1 coding gene *PDCD1 *[222] or TGF- β signal transduction in CAR-T cells has been used to suppress the immunosuppressive pathway [223]. Moreover, the fourth-generation CAR possesses enhanced T cell function, which can secrete additional anti-tumor cytokines when activated [224]. The fifth-generation CAR comprises the addition of the IL-2 receptor domain between the CD3 and CD28 signal regions in the extracellular domain [225]. In addition to optimizing CAR-T cell design, as mentioned above, another strategy is to combine ACT with other therapies. Combining multiple immune interventions is necessary to reverse “cold” tumors [197].

For example, vesicular stomatitis virus (VSV) and reovirus were used in mice with normal immune function, revealing that tumor pretreatment with OVs had both beneficial and harmful consequences on CAR-T cells. Although cytokines and chemokines are produced after OV infection, CAR-T cells simultaneously undergo depletion due to cascade inflammation induced by type I IFN [226]. OVs were loaded into CAR-T cells as an alternative to combination therapy in an attempt to decrease the unpredictable adverse reactions caused by the combination of OVs and CAR-T cells. CAR-T cell therapy loaded with OVs was highly valid in curing solid tumors in mice after intravenous administration with subtle toxicity [187].

Engineering adoptive cell genes decrease the toxicity associated with systemic antibody administration [227], while the low targeting efficiency of DNA transfection limits the application of multi-genomic engineering in T cells [228]. Researchers may consider investigating a new construction or combination as a more appropriate treatment strategy.

### Selection of the right drug or biotechnology to enhance certain cellular therapy

Several combinations of adoptive cell therapies based on CAR-T cells and different immunotherapies have been introduced, including ICIs/ monoclonal antibody drugs, small molecule inhibitors, cytokine drugs, PROTACs, and OVs. The “cold” tumors can be “heated” by combined therapies and transformed into immunoreactive phenotypes [229]. Optimizing multi-agent cancer immunotherapy combination regimens remains a focal point in tumor immunity [230]. The FDA has approved some combination strategies of immunotherapy, including multi-ICIs (such as lpilimumab combined with nivolumab for BRAF^wt^ metastatic melanoma) [231], ICI combined with chemotherapy (such as pembrolizumab combined with pemetrexed for NSCLC) [232], and ICI combined with targeted therapy (such as pembrolizumab combined with axitinib for renal cell carcinoma) [231, 233]. Treatment selection will likely be harder with the emergence of numerous types of cellular therapy. DCs are the most active and powerful full-time APCs in the human body, also known as “scouts” of anti-tumor immunity [234]. CAR-NK and CAR-M are considered promising cell types for the treatment of solid tumors [235], and CAR-NK presents incredible advantages due to the higher clinical safety, the existence of CAR-independent killing mode, and the reduced risk of GVHD [236–238]. Erythrocytes constitute the majority of blood cells in human blood and have a long circulating half-life, high biocompatibility, and safe elimination mechanism, which hints about their potential role as drug carriers [239]. Since the concept of “red blood cell therapy” was first put forward by Rubius in 2014, trials on the application of modified red blood cells in cancer treatment have been explored. However, there are no criteria for selecting drugs or biotechnology to enhance cellular therapy.

The biggest challenge in developing these high-level combinations may be to find a system to identify the most appropriate combinations so that only the most promising combinations undergo clinical trials.

### Future perspectives

The continuous development of efficient and low-toxicity cellular therapy facilitates the whole immune process and relies on genetic engineering, cell reprogramming, and synthetic biology. Meanwhile, vigorously expanding the clinical application of the previously approved products to other indications should also be explored. Considering the high cost of cellular therapy, widespread applications in medical institutions are limited, and patients might be unwilling to receive the treatment. Therefore, efforts must be brought to improve the clinical transformation and commercialization of cellular therapy. Improvements in manufacturing processes (such as decentralized manufacturing to expand the global scale and spot products of allogeneic therapy) will likely assist in the adoption of cellular therapy. Furthermore, the regulation of ACT products should be strengthened, and the labels of approved products should be kept up to date, including warnings and precautions, adverse reactions, patient counseling information, etc. As more clinical trials are performed in this field, a large number of patients will benefit from enhanced cellular therapy.

## Conclusion

This review is the first to summarize the novel concept of enhanced cellular therapy and discusses how various drugs or biotechnology enhance cellular therapy, to improve treatment strategies for cancer patients, especially solid tumor patients. We firmly believe that enhanced cellular therapy will play a powerful role in tumor immunotherapy in the future.

## Data Availability

All data generated or analysed during this study are included in this published article and referenced articles are listed in the References section.

## Abbreviations

ACT: Adoptive cellular therapy
AICD: Activation-induced cell death
ALL: Acute lymphoblastic leukemia
APC: Antigen-presenting cell
AR: Androgen receptor
B-ALL: B-cell acute lymphoblastic leukemia
BCMA: B cell mature antigen
BD: Bromine domain
BiTE: Bispecific T cell conjugate
BRD: Bromine domain protein
BRDT: Testis-specific BRD
BTK: Bruton’s tyrosine kinase
BTLA: B and T lymphocyte attenuator
CAR: Chimeric antigen receptor
CAR-M: Chimeric antigen receptor macrophages
CAR-NK: Chimeric antigen receptor natural killer
CBL-B: Casitas B-lineage lymphoma proto-oncogene B
CCR4: CC-chemokine receptor 4
ccRCC: Clear cell renal cell carcinoma
CIK: Cytokine-induced killer
CRBN: Cereblon
CRPC: Castration-resistant prostate cancer
DAMP: Damage-associated molecules pattern
VML: Chronic myeloid leukemia
CR: Complete remission
CRS: Cytokine release syndrome
CSF: Colony-stimulating factor
CSF-1R: Colony-stimulating factor 1 receptor
CSR: Chimeric switch receptor
CTLA 4: Cytotoxic T lymphocyte-associated protein 4
CXCR: C-X-C chemokine receptor
DC: Dendritic cell
DIC: Disseminated intravascular coagulation
DLBCL: Diffuse large B-cell lymphoma
ER: Estrogen receptor
FDA: The United States Food and Drug Administration
G-CSF: Granulocyte-colony-stimulating factor
GM-CSF: Granulocyte macrophage-colony-stimulating factor
GvHD: Graft-versus-host disease
HIF1 α: Hypoxia-inducible factor 1 subunit alpha
HSC: Hematopoietic stem cell
IAP: Inhibitor of apoptosis protein
ICANS: Immune effector cell-associated neurotoxicity syndrome
ICD: Immunogenic cell death
ICI: Immune checkpoint inhibitor
IFN: Interferon
IgSF: Immunoglobulin superfamily
IL: Interleukin
LAG: Lymphocyte activation gen
LAK: Lymphokine-activated killer
LILRB: Leukocyte Ig-like receptor subfamily B
meso-CAR-T: Mesothelin-redirected CAR-T
MetAP-2: Methionine aminopeptidase 2
MDM2: Mouse double minute 2
MDR: Multidrug resistance
MDSC: Marrow-derived suppressor cell
NI: Neuroinflammation
NK: Natural killer
OV: Oncolytic virus
PAMP: Pathogen-associated molecular pattern
PBMC: Peripheral blood mononuclear cell
PD-1: Programmed cell death protein 1
PDA: Pancreatic ductal adenocarcinoma
PDT: Photodynamic therapy
PPI: Protein–protein interaction
PROTAC: Proteolysis targeting chimera
PTD: Protein targeted degradant
ROR1: Receptor tyrosine kinase-like orphan receptor 1
SARM: Non-steroidal androgen receptor ligand
scFv: Single-chain fragment variable
Siglec-7: Sialic acid-binding immunoglobulin-like lectin 7
SIRPα: Signal regulatory protein alpha
TAA: Tumor-associated antigen
TAM: Tumor-associated macrophages
TCR-T: T cell receptor-gene engineered T
TIGIT: T cell Ig and ITIM domain
TIL: Tumor-infiltrating lymphocyte
TIM-3: T cell immunoglobulin and mucin domain-containing protein 3
TME: Tumor microenvironment
TNF: Tumor necrosis factor
Treg: Regulatory T cell
TSA: Tumor-specific antigen
VHL: Von Hippel-Lindau
VISTA: V-domain Ig suppressor of T-cell activation
VSV: Vesicular stomatitis virus
UPS: Ubiquitin–proteasome system
